# Systematic Performance Evaluation of WebGazer Webcam-Based Eye Tracking with Spatial and Angular Error Analysis

**DOI:** 10.3390/jemr19050099

**Published:** 2026-09-08

**Authors:** Evgenia Psarra, Vassilios Krassanakis, Anastasios L. Kesidis

**Affiliations:** 1 Department of Surveying & Geoinformatics Engineering, University of West Attica, Egaleo Park Campus, Ag. Spyridonos Str., Egaleo, 12243 Athens, Greece; krasvas@uniwa.gr (V.K.); akesidis@uniwa.gr (A.L.K.); 2 Department of Informatics, University of Piraeus, Karaoli & Dimitriou 80, 18534 Piraeus, Greece

**Keywords:** eye tracking, WebGazer, webcam-based eye tracking, spatial and angular gaze error metrics, performance evaluation

## Abstract

Eye tracking technologies have become increasingly accessible through webcam-based solutions such as WebGazer, enabling large-scale and low-cost experimentation. However, their performance remains sensitive to several experimental and device-related parameters. This study presents a controlled experimental framework developed to systematically evaluate WebGazer under configurable experimental conditions. Within this framework, we design and implement a controlled experimental protocol to investigate the effect of key parameters, including (i) joint calibration/evaluation point-density configuration, (ii) evaluation-point distribution, (iii) target presentation duration, (iv) stimulus color, and (v) stimulus (target) size. WebGazer’s performance is assessed using both device-pixel spatial error metrics and angular error (delta), providing complementary measures of gaze accuracy. Results from 20 participants show that denser point configurations were associated with lower mean spatial error, from 1383 to 1009 device pixels, and lower mean angular error, from 13.556 to 10.322 degrees, within the tested conditions. Across the aggregated condition summaries, the joint point-density configuration showed the widest spatial-error range, whereas target presentation duration showed the widest angular-error range. Taken together, these two factors displayed the clearest descriptive differences, followed by evaluation-point distribution. Pseudo-random layouts were associated with moderately higher errors than fixed layouts. In contrast, visual properties such as color and size showed comparatively smaller and less consistent differences, with stimulus size displaying a non-monotonic pattern. Overall, temporal and calibration-related settings showed the clearest descriptive associations with WebGazer performance, while visual design choices showed smaller differences. This study provides a practical quantitative framework for systematically assessing webcam-based eye tracking systems under realistic usage conditions.

## 1. Introduction

Eye tracking has traditionally depended on specialized hardware, such as infrared-based systems, which can provide highly accurate measurements but are often costly and require controlled laboratory environments. As a result, their use has been largely limited to small-scale, in-lab studies. However, in recent years advances in computer vision and web technologies have made it possible to perform eye tracking using standard webcams. This shift has made eye tracking research more accessible, significantly lowering costs and allowing experiments to be conducted with larger and more diverse groups of participants in more natural settings [1,2,3]. Although webcam-based methods are generally less precise than dedicated devices, they have proven to be sufficiently reliable for many applications, such as usability studies, reading behavior analysis, and large-scale online experiments [4,5]. Overall, their ease of use and scalability have made them an increasingly attractive option for researchers in human–computer interaction and behavioral sciences.

Within this context, several tools have been developed to leverage these capabilities, with WebGazer [3] being one of the most widely used approaches. WebGazer offers several practical advantages, including low cost, ease of use, and the ability to support experiments with a large number of participants without requiring specialized eye-tracking hardware. In particular, it is an online eye tracker that uses common webcams, already present in mobile devices, to infer the eye-gaze locations of web visitors on a page in real time. Hutt et al. [6] conducted a study on text comprehension exercises among students using the WebGazer tool. Semmelmann and Weigelt [5] used WebGazer with consumer-grade webcams to investigate the potential of online eye tracking to benefit from the common advantages of online data collection. They compared three in-lab-conducted tasks (fixation, pursuit, and free viewing) with online-acquired data to analyze the spatial precision in the first two, and replicability of well-known gazing patterns in the third task. Yang et al. [7] studied the incorporation of WebGazer into the JavaScript library jsPsych [8] used by behavioral researchers, and adjusted the procedure and code to reduce the calibration/validation phase and improve the temporal resolution. Additionally, Steffan et al. [9] report a multi-lab study where they compared the remote web-based eye tracking with in-lab eye tracking in young children. In their study, they utilized WebGazer and jsPsych. Results of their remotely tested sample revealed that web-based eye tracking successfully captured the goal-based action predictions.

Beyond the development of eye tracking tools, an equally important research direction focuses on the evaluation of their performance, particularly in terms of accuracy and reliability. This is especially relevant for low-cost and webcam-based solutions, where measurement noise and experimental conditions can significantly affect the quality of the recorded gaze data. The evaluation of gaze data quality has attracted significant attention, especially in the context of low-cost eye tracking systems. Ooms and Krassanakis [10] and Ooms et al. [11] examined the accuracy and precision of gaze data by comparing recorded fixation locations with known reference points, using both artificial and human eye measurements. Their approach includes noise modeling and error analysis techniques [10], which are closely related to established post-processing tools such as the EyeMMV toolbox [12]. EyeMMV provides practical methods for detecting fixation events based on spatial dispersion criteria, making it a useful framework for analyzing gaze data, while its underlying algorithm is also used in fixation detection approaches adopted in such evaluation studies. This connection highlights the importance of combining reliable analysis tools with systematic evaluation procedures when assessing eye tracking performance.

Evaluating the performance of eye tracking systems is a key topic in the literature, with most studies focusing on metrics such as accuracy and precision. In particular, several works have examined the reliability of low-cost devices by comparing their outputs either with high-end systems or with known ground truth data. Previous studies have proposed methodological approaches for estimating spatial error and handling noise in gaze measurements, while others have explored how different experimental conditions influence performance. Overall, these findings suggest that, although webcam-based and low-cost solutions are generally less accurate than dedicated hardware, they can still provide sufficiently reliable data for many practical applications, especially when appropriate calibration and evaluation procedures are applied.

In addition to webcam-based eye tracking, alternative low-cost approaches such as mouse tracking have been explored for studying visual behavior. For example, the MatMouse toolbox [13] supports experimental studies based on cursor movement analysis, offering a complementary perspective on user interaction patterns. These approaches highlight a broader trend toward scalable and cost-effective methods for capturing behavioral data. In another eye-tracking application, Brandl et al. [14] studied the additional information provided by gaze data, such as total reading times, gaze entropy, and decoding accuracy, while comparing the implementation of the WebQAmGaze dataset on the language models mBERT, distil-mBERT, XLMR, and XLMR-L for three European languages.

Despite the growing adoption of webcam-based eye tracking systems, several important challenges remain. One of the main limitations is the lack of large-scale, reliable ground truth data, which are essential for understanding gaze behavior and for accurately evaluating system performance under realistic conditions. Collecting such data outside controlled laboratory environments remains a complex and resource-intensive process. In addition, existing studies often rely on isolated experimental setups, making it difficult to directly compare results across different configurations. This highlights the absence of unified platforms or applications that enable systematic and reproducible evaluation of eye tracking performance. At the same time, prior research has demonstrated that various experimental parameters can significantly influence the accuracy and reliability of eye tracking systems, particularly in low-cost and webcam-based settings. Factors such as calibration density, spatial distribution of calibration and evaluation points, stimulus duration, and visual characteristics have been shown to affect gaze estimation accuracy, either by modifying the experimental conditions or by introducing additional variability. However, these parameters are often studied independently, and there is limited work that investigates their combined impact within a consistent experimental framework. This gap motivates the need for a more systematic and unified approach to evaluating these factors.

To address this gap, this paper presents a comprehensive, multi-parameter evaluation framework designed to systematically investigate WebGazer performance under controlled and configurable conditions. The primary contribution of this study lies in the controlled one-factor-at-a-time manipulation of five experimental factors within the same participant group and software framework. The aim is not to claim a full factorial analysis of all possible parameter interactions, but to provide a reproducible framework and a participant-level quantitative comparison of selected experimental settings. To make the objectives explicit, the following research questions guide the analysis: RQ1: How is the joint calibration/evaluation point-density configuration associated with WebGazer spatial and angular error? RQ2: How do fixed and pseudo-random evaluation-point distributions compare in terms of gaze-estimation error? RQ3: How does recorded error vary across target presentation durations? RQ4: To what extent do recorded spatial and angular errors vary across stimulus colors and sizes? RQ5: Which examined parameter displays the largest descriptive differences across participants?

The remainder of this paper is organized as follows. Section 2 summarizes related work and recent state-of-the-art developments. Section 3 describes the experimental design, workflow, evaluation metrics, participant-level descriptive evidence, and participants/equipment used in the study. Section 4 presents the experimental results. Section 5 discusses the findings and limitations of the study. Finally, Section 6 concludes the paper and outlines directions for future work.

## 2. Related Work and State of the Art

Recent eye tracking literature emphasizes that the field has expanded from laboratory-based infrared systems toward more accessible and heterogeneous sensing approaches, including webcam-based, model-based, appearance-based, electrooculography-based, and video-oculography-based techniques [15]. Recent review-oriented work also highlights the importance of evaluation dimensions such as accuracy, latency, robustness, device constraints, and cross-platform reproducibility [15]. In parallel, bibliometric evidence shows a sustained increase in eye-tracking applications across educational technology and human–computer interaction, further motivating the need for transparent and reproducible evaluation procedures [16].

Within webcam-based eye tracking, WebGazer and related JavaScript-based experimental workflows have enabled scalable remote data collection, but they also introduce important methodological challenges. Performance can depend on calibration design, spatial layout, target timing, display geometry, camera placement, lighting, and participant behavior. Prior studies have validated the feasibility of webcam-based eye tracking for specific tasks and populations [3,4,5,6,7,8,9], while studies on low-cost eye tracking and gaze-data quality have provided methods for interpreting spatial error, noise, and fixation accuracy [10,11,12]. However, comparatively less attention has been given to a unified parameter-sensitivity evaluation of WebGazer itself under controlled, configurable settings. The present work addresses this gap by examining multiple setup parameters within the same experimental framework and by reporting both aggregated results and participant-level descriptive evidence.

## 3. Materials and Methods

### 3.1. Experimental Design and Parameters

The experimental setup was guided by the need to systematically evaluate the impact of key experimental parameters on WebGazer performance under controlled conditions. The environment was configured to support real-time gaze estimation and flexible parameter manipulation, enabling the systematic variation in calibration density, spatial distribution, target presentation duration, color, and size.

The experimental design is based on the systematic variation in key parameters that are expected to influence eye tracking performance. These parameters, along with their corresponding units and tested values, are summarized in Table 1.

In the point-density analysis, calibration and evaluation density were varied jointly through matched configurations (5-5, 9-9, 12-12, and 18-18). Consequently, the results for this factor represent the combined calibration/evaluation point-density configuration and should not be interpreted as isolating either density component. The study followed a one-factor-at-a-time repeated-measures design. Each examined parameter was varied across its predefined levels while the remaining parameters were held at the reference values reported in the corresponding Results subsection. The study should therefore be interpreted as a controlled descriptive parameter-sensitivity analysis rather than as a full factorial experiment.

The systematic variation in these parameters enables a controlled descriptive comparison of the tested WebGazer configurations.

Each participant completed 19 calibration-evaluation runs. Table 2 presents the corresponding 19 distinct run configurations and repeats the common reference configuration (Run 1) in the final displayed row solely to make its role in the factor-wise comparisons explicit. The repeated final row does not represent an additional experimental run. The matrix is grouped by analytical factor rather than chronological order because the original trial-order log was not preserved.

The 18-18/fixed/5 s/blue-green/20-20 configuration served as the reference for the point-density, distribution, duration, and size comparisons. The color comparison used matched calibration and evaluation colors. A new calibration sequence was completed at the beginning of every run. The retained records do not document a formal randomized or counterbalanced sequence, and consequently, practice, fatigue, and order effects cannot be excluded.

### 3.2. Experimental Workflow

The experimental workflow consists of four main stages. First, the participant provides the active screen dimensions, screen resolution, and eye-to-screen-center distance required for the angular calculations. Second, a new calibration sequence is completed for the selected run configuration. Third, the evaluation stage presents the target sequence while WebGazer returns gaze predictions. Finally, each prediction is paired with the target visible at that time and the spatial and angular error metrics are calculated. One trial denotes one complete calibration-evaluation run under one of the 19 distinct configurations shown in Table 2. The final repeated reference row is included only for analytical presentation.

During the calibration stage, WebGazer is trained through a sequence of predefined calibration points. The participant successively clicks the displayed calibration targets using the selected point-density configuration (5, 9, 12, or 18 points). Each target requires one click and disappears immediately after that click, after which the next target appears. Target size was specified in browser/CSS pixels and could be set to 10, 12, 14, 16, 18, or 20 CSS pixels. The target color could be selected from blue, red, magenta, black, green, and brown. A new calibration sequence was initiated at the beginning of every calibration-evaluation run.

After calibration, the evaluation stage begins. Participants follow a circular target while keeping their head as still as possible. Each target remains visible for 2, 3, 4, or 5 s, according to the selected condition, and is immediately replaced by the next target. Evaluation sequences contain 5, 9, 12, or 18 targets. Every gaze prediction returned by WebGazer while a target was visible was associated with that target and included in the corresponding error calculation after application of the stated within-run exclusion rule. The retained implementation notes do not document additional temporal down-sampling or smoothing. No separate validation stage was performed. The evaluation sequence itself was used to assess prediction error.

The evaluation process also includes pseudo-random target positions. In this configuration, each evaluation target is shifted within a 14% range around its corresponding fixed position, preserving the overall spatial structure while introducing controlled positional variability. This condition enables a descriptive comparison with the fixed layout, and it does not by itself identify the mechanism responsible for any observed difference.

#### WebGazer Implementation Details

GazerApp, a browser application developed for this study, integrated the 2016 version of WebGazer.js, a client-side browser library. Each calibration target required one click, and all WebGazer predictions returned while the current evaluation target was visible were paired with that target. No separate validation stage was used. The study was conducted in accordance with applicable personal data protection requirements and the research ethics principles of the University of West Attica. Under Article 279.2.a of Act 4957/2022 and the Research Ethics and Conduct Regulation of the University of West Attica, submission to the Research Ethics Committee was not mandatory for this non-funded, low-risk technical evaluation, which did not raise serious or complex ethical issues. Before the experiment, each participant was informed about its purpose and procedure and provided consent to participate. Only estimated gaze coordinates, target coordinates, and derived error metrics were recorded. No directly identifiable or sensitive personal data were collected, and the procedure involved no medical intervention.

### 3.3. Evaluation Metrics

Both the target position rendered by the browser and the gaze prediction returned by WebGazer were recorded in native device pixels. Chrome browser zoom was fixed at 100%. With Windows display scaling set to 300%, the effective CSS-to-device conversion factor was d = 3.0, so one CSS pixel corresponded to three native device pixels along both axes. For every target point T and predicted gaze point P, both coordinate pairs were converted identically as x_device = d * x_CSS and y_device = d * y_CSS. The Euclidean target-prediction distance TP was then calculated in device pixels. This scale-adjusted device-pixel distance is the spatial prediction error reported in Table 3, Table 4, Table 5, Table 6 and Table 7. At the end of each participant-condition run, mean, minimum, maximum, and standard-deviation summaries were calculated. Errors exceeding three times the standard deviation of the values within the corresponding participant-condition run were excluded before the run-level summaries were computed. The criterion was not applied globally across the complete dataset.

For angular-error calculation, the native device-pixel coordinates were converted separately to physical screen coordinates. Let W = 28.8 cm and H = 18.0 cm denote the active display width and height, and let R_x = 3840 and R_y = 2400 denote the native horizontal and vertical device-pixel resolutions. The physical scale factors were s_x = W/R_x = 0.0075 cm per device pixel and s_y = H/R_y = 0.0075 cm per device pixel. At d = 3.0, the corresponding scale is 0.0225 cm per CSS pixel in both directions. Consequently, px in Table 3, Table 4, Table 5, Table 6 and Table 7 denotes device pixels, whereas the target-diameter settings refer to CSS pixels.

For any CSS-coordinate point Q = (x_Q,CSS, y_Q,CSS), the corresponding device-pixel point is Q_device = (3 * x_Q,CSS, 3 * y_Q,CSS). Target point T, prediction point P, and screen center K were expressed in this same device-pixel coordinate system before the distances KT, KP, and TP were converted to centimeters using s_x and s_y. Point A is defined on the normal to the screen plane through K, with AK equal to the measured eye-to-screen-center distance of 41 cm. Thus, KT, KP, TP, AT, AP, and AK are expressed in centimeters for the angular calculations; spatial error is reported in device pixels, and angular error is reported in degrees. Windows display scaling was therefore handled once, through the CSS-to-device conversion, before the native-resolution scale factors were applied. All values in Table 3, Table 4, Table 5, Table 6 and Table 7 are in device pixel or in degrees.

**Figure 1:**
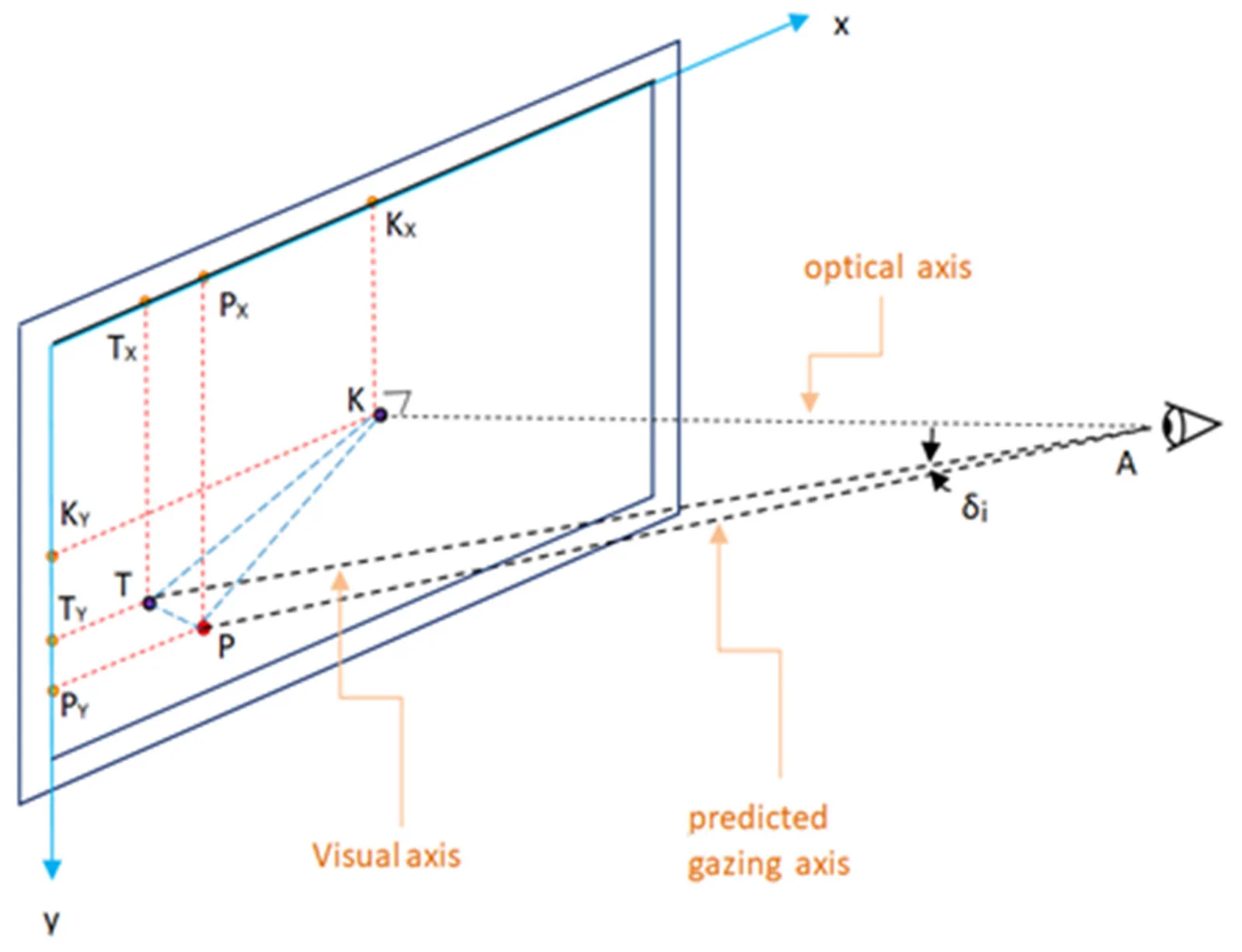
Setup geometry and evaluation metrics.

For the observation-level angular metric, a deviation angle δ_i_, as illustrated in Figure 1, is calculated for every target-prediction pair. KT, KP, and TP are first expressed in centimeters using the conversion described above. AT and AP are then obtained from the right triangles AKT and AKP, and δ_i_ is calculated from triangle ATP using the law of cosines:

(1) $$\delta_{i}=\arccos\left(\frac{{AT}^{2}+{AP}^{2}-{TP}^{2}}{2\cdot AT\cdot AP}\right)$$

The overall angular error delta is the mean of the observation-level delta_i values. The delta metric is used as the primary angular error measure because it is calculated for each target-prediction pair before aggregation. Participant-level descriptive summaries underlying Table 3, Table 4, Table 5, Table 6 and Table 7 are provided in Appendix A. Across the aggregated condition summaries, the largest descriptive ranges are observed for target presentation duration and joint point-density configuration. Point density shows the wider device-pixel range, whereas duration shows the slightly wider angular range. The fixed versus pseudo-random contrast is smaller, and the color and size ranges are smaller still.

### 3.4. Participants, Experimental Trials, and Equipment

Twenty adult volunteers participated in the experimental study. All participants were positioned 41 cm from the center of the screen. In total, 380 calibration-evaluation runs were recorded, with each of the 20 participants completing 19 actual runs under the distinct configurations summarized in Table 2. The study focused on anonymous gaze-performance measurements rather than demographic profiling. Therefore, age, gender, visual correction status, and previous eye-tracking experience were not retained in the records. All data underlying Table 3, Table 4, Table 5, Table 6 and Table 7 were collected on a Dell XPS 13 Plus 9320 laptop equipped with a 13.4-inch display. The active display area, excluding bezels, was 28.8 cm × 18.0 cm. Its integrated HD RGB camera captured video at 1280 × 720 pixels and 30 fps. GazerApp was run in Google Chrome on Windows 11 Pro, under stable indoor lighting, with the integrated webcam positioned above the display and facing the participant.

## 4. Results

Results are organized by the parameter under examination. For each condition, the analysis reports scale-adjusted device-pixel spatial-error and angular-error summaries, including the mean, maximum, minimum, and standard deviation. The comparisons are descriptive. Participant-level values are provided in Appendix A.

### 4.1. Effect of Calibration and Evaluation Point Density

In Table 3, the final average values from the experimental trials of all participants are presented according to the number of calibration and evaluation points. These values were obtained from experimental trials where the following parameters were kept fixed:

- Width of the calibration target: 20 px
- Diameter of the evaluation target: 20 px
- Target presentation duration: 5 s
- Distribution of evaluation points: Fixed
- Color of the calibration target: blue
- Color of the evaluation target: green

Table 3 shows a descriptive association between the joint calibration/evaluation point-density configuration and the recorded errors. Within the tested configurations, denser layouts correspond to lower mean spatial and angular errors. Across the full tested range, mean spatial error decreases from 1383 device pixels in the 5-5 condition to 1009 device pixels in the 18-18 condition, a difference of 374 pixels (27.0%), while mean angular error decreases from 13.556 to 10.322 degrees, a difference of 3.234 degrees (23.9%). The largest adjacent reduction occurs from 9-9 to 12-12: mean spatial error is 186 device pixels lower (15.2%), and mean angular error is 1.737 degrees lower (14.2%). From 12-12 to 18-18, the corresponding differences are smaller, at 28 device pixels and 0.213 degrees. The minimum spatial error decreases from 97 device pixels in the 5-5 condition to 58 device pixels in the 18-18 condition, while the standard deviation is lower in the 12-12 and 18-18 configurations than in the two sparser configurations.

These aggregate summaries show a monotonic reduction in mean error across the denser joint point-density configurations. The comparison concerns the joint point-density configuration and does not separate calibration density from evaluation density.

### 4.2. Effect of Evaluation Point Distribution

Table 4 presents the final average values from the experimental trials of all participants according to the fixed or pseudo-random configuration of the evaluation points. These values were obtained from experimental trials where the following parameters were kept fixed:

- Width of the calibration target: 20 px
- Diameter of the evaluation target: 20 px
- Target presentation duration: 5 s
- Color of the calibration target: blue
- Color of the evaluation target: green
- Number of calibration points: 18
- Number of evaluation points: 18

In Table 4, the pseudo-random configuration is associated with higher mean spatial and angular errors than the fixed configuration in this sample. This is a descriptive contrast and does not establish that reduced predictability caused the difference. Reduced spatial predictability is considered one possible explanation.

The minimum spatial error is 96 device pixels in the pseudo-random condition and 58 device pixels in the fixed condition, while the maximum values are 3121 and 2989 device pixels, respectively. The spatial-error standard deviation is also higher in the pseudo-random condition (666 versus 617 device pixels). The mean differences follow the same direction: 1160 versus 1009 device pixels for spatial error and 11.886 versus 10.322 degrees for angular error. These descriptive contrasts do not establish that reduced predictability caused the difference.

### 4.3. Effect of Target Presentation Duration

In Table 5, the final mean values from the experimental trials of all participants are presented according to target presentation duration. These values were obtained from experimental trials where the following parameters were kept fixed:

- Width of the calibration target: 20 px
- Diameter of the evaluation target: 20 px
- Distribution of evaluation points: Fixed
- Color of the calibration target: blue
- Color of the evaluation target: green
- Number of calibration points: 18
- Number of evaluation points: 18

Table 5 shows that shorter target presentation durations are associated with higher mean spatial and angular errors in the tested sample. Mean spatial error ranges from 1334 device pixels at 2 s to 1009 device pixels at 5 s, while mean angular error ranges from 13.741 to 10.322 degrees. These values describe the observed conditions and do not constitute a formal repeated-measures test. The standard-deviation summaries are similar across durations and do not support a claim that the shortest duration produced the greatest variability.

### 4.4. Effect of Stimulus Color

In Table 6, the final mean values from the experimental trials of all participants are presented according to the color of the calibration targets and evaluation targets. These values were obtained from experimental trials where the following parameters were kept fixed:

- Width of the calibration target: 20 px
- Diameter of the evaluation target: 20 px
- Target presentation duration: 5 s
- Distribution of evaluation points: Fixed
- Number of calibration points: 18
- Number of evaluation points: 18

Across the aggregated condition summaries, differences across colors are comparatively small. Mean spatial error ranges from 956 device pixels for Black/Black to 1041 device pixels for Green/Green, and mean angular error ranges from 9.813 to 10.681 degrees. These ranges are smaller than those observed across duration and point-density conditions, so color is treated as a secondary setting in the present sample rather than as a demonstrated effect.

### 4.5. Effect of Stimulus Size

In Table 7, the final mean values from the experimental trials of all participants are presented according to the size of the calibration and evaluation targets. These values were obtained from experimental trials where the following parameters were kept fixed:

- Target presentation duration: 5 s;
- Distribution of evaluation points: Fixed
- Color of the calibration target: blue
- Color of the evaluation target: green
- Number of calibration points: 18
- Number of evaluation points: 18

Table 7 shows small, non-monotonic differences across target sizes. The lowest mean spatial and angular errors both occur at 12/12 (968 device pixels and 9.922 degrees), with 14/14 yielding the next-lowest mean values. The results do not show a consistent advantage for either larger or smaller targets across the tested range, supporting a cautious interpretation of target size as a secondary setting in this dataset.

## 5. Discussion

This study examines how recorded WebGazer error varies across selected experimental configurations using device-pixel spatial and angular metrics. In the present sample, target presentation duration and the joint calibration/evaluation point-density configuration show the largest descriptive differences, followed by evaluation-point distribution. Their relative ranking depends on the metric: point density has the wider mean device-pixel range, whereas duration has the slightly wider mean angular range. Shorter target presentation durations are associated with higher recorded errors. One possible explanation is that participants have less time to stabilize their gaze before the next target appears. Longer intervals correspond to lower mean errors in the present data, but this interpretation requires confirmation through a design that controls trial order and applies predefined repeated-measures analysis.

Denser joint calibration/evaluation point configurations are also associated with lower spatial and angular errors. Such configurations may provide broader screen coverage, but they also lengthen the procedure and may increase participant burden. Because calibration and evaluation density were varied together, their separate contributions cannot be determined from the present data. The pseudo-random distribution is associated with somewhat higher errors than the fixed distribution. Reduced predictability is one plausible explanation, but it was not directly measured and should not be treated as an established cause. Fixed layouts may support more regular transitions, whereas pseudo-random layouts may better represent less predictable interaction scenarios. The appropriate choice therefore depends on the intended use of the experiment. Stimulus color and size show smaller and less consistent descriptive differences. Color values vary within a narrow range, and errors are non-monotonic: both mean-error minima occur at 12/12, with 14/14 yielding the next-lowest values. These parameters should therefore be considered in relation to display type, contrast, lighting, and participant characteristics rather than interpreted as having a uniform effect. Overall, webcam-based eye tracking remains accessible and scalable, but the observed measurements are sensitive to experimental setup and implementation choices. The proposed framework supports structured comparison of selected settings under a common protocol. At the same time, its one-factor-at-a-time design supports descriptive within-sample comparisons only and does not establish interactions or causal effects among the tested parameters.

This study has several limitations. First, head movement, camera positioning, lighting, and participant-specific differences were not controlled with strict physical constraints. Second, all data were collected using one laptop, integrated webcam, browser, and operating system, limiting device-level generalization. Third, the study was exploratory and demographic covariates were not retained. Fourth, calibration and evaluation density were varied jointly. Fifth, the original chronological trial-order log was not preserved, and no formal randomization or counterbalancing schedule can be verified. Additionally, practice, fatigue, and order effects may therefore have contributed to condition differences. Finally, the reported mean values may nevertheless provide an indicative reference for studies using comparable settings, provided that these methodological limitations are taken into account. Spatial error expressed in device pixels is specific to the tested native resolution and display-scaling configuration. Cross-device comparisons should therefore use physical or angular units and explicitly account for the browser’s CSS-to-device-pixel mapping. The effects of changing native resolution, Windows scaling, or browser zoom were not experimentally evaluated.

## 6. Conclusions

This study presents a quantitative, one-factor-at-a-time evaluation of WebGazer across five experimental factors: joint calibration/evaluation point-density configuration, evaluation-point distribution, target presentation duration, stimulus color, and stimulus size. Spatial error in scale-adjusted device pixels and observation-level angular error are reported within the same participant group and browser-based framework.

Within the present sample, the largest descriptive differences occur across target presentation-duration conditions and joint point-density configurations. Their ranking depends on the metric: point density has the wider mean device-pixel range, whereas duration has the slightly wider mean angular range. Longer durations and denser configurations are associated with lower mean errors, while the pseudo-random layout is associated with somewhat higher errors than the fixed layout. These associations do not establish causality or population-level effects. Stimulus color and size show smaller and less consistent differences, with size displaying a non-monotonic pattern. The findings identify settings that warrant consideration and confirmatory evaluation when designing webcam-based eye-tracking studies.

Future work should use larger samples and full or partial factorial designs with predefined repeated-measures analyses. Studies should retain the complete chronological trial schedule, randomization or counterbalancing information. Additional experiments should compare hardware, browsers, webcams, and display sizes and should consider more controlled setups, such as a chin rest. Although the present approach is preliminary and exploratory, it provides a quantitative framework that can be refined alongside established gaze-data quality methods, including those discussed by Ooms and Krassanakis [10], and applied in future usability and human–computer interaction research.

## Figures and Tables

**Table 1:** Experimental parameters, units, and tested values used in the evaluation framework.

Parameter	Description	Values Tested
Calibration Density	Number of calibration points	5, 9, 12, 18
Evaluation Density	Number of evaluation points	5, 9, 12, 18
Distribution	Spatial arrangement of points	Fixed, Pseudo-random
Target Presentation Duration	Time each target is displayed	2, 3, 4, 5 s
Target Point Color	Color of dots	Black, blue, brown, green, magenta, red
Target Point Size	Size of dots	10, 12, 14, 16, 18, 20 px

**Table 2:** Reconstructed experimental-condition matrix for the 19 completed runs. The final row (1 *) repeats the reference condition (Run 1) for analytical purposes and does not represent an additional experimental run. Rows are grouped by analytical factor rather than chronological order.

Run	Analytical Block	Cal./Eval. Points	Evaluation Distribution	Duration (s)	Cal./Eval. Color	Cal./Eval. Diameter (px)
1	Reference	18/18	Fixed	5	Blue/Green	20/20
2	Point density	5/5	Fixed	5	Blue/Green	20/20
3	Point density	9/9	Fixed	5	Blue/Green	20/20
4	Point density	12/12	Fixed	5	Blue/Green	20/20
5	Distribution	18/18	Pseudo-random	5	Blue/Green	20/20
6	Duration	18/18	Fixed	2	Blue/Green	20/20
7	Duration	18/18	Fixed	3	Blue/Green	20/20
8	Duration	18/18	Fixed	4	Blue/Green	20/20
9	Color	18/18	Fixed	5	Black/Black	20/20
10	Color	18/18	Fixed	5	Blue/Blue	20/20
11	Color	18/18	Fixed	5	Brown/Brown	20/20
12	Color	18/18	Fixed	5	Green/Green	20/20
13	Color	18/18	Fixed	5	Magenta/Magenta	20/20
14	Color	18/18	Fixed	5	Red/Red	20/20
15	Size	18/18	Fixed	5	Blue/Green	10/10
16	Size	18/18	Fixed	5	Blue/Green	12/12
17	Size	18/18	Fixed	5	Blue/Green	14/14
18	Size	18/18	Fixed	5	Blue/Green	16/16
19	Size	18/18	Fixed	5	Blue/Green	18/18
1 *	Repeated reference	18/18	Fixed	5	Blue/Green	20/20

**Table 3:** Aggregated WebGazer performance according to the number of calibration and evaluation points. For instance, 5-5 denotes the use of 5 points in both the calibration and evaluation processes.

Evaluation Metrics	5-5	9-9	12-12	18-18
mean spatial error (px)	1383	1223	1037	1009
maximum spatial error (px)	3742	3574	2917	2989
minimum spatial error (px)	97	83	69	58
standard deviation of spatial error (px)	797	725	598	617
mean delta error (deg)	13.556	12.272	10.535	10.322
maximum delta error (deg)	37.251	35.709	29.792	30.482
minimum delta error (deg)	0.959	0.836	0.705	0.591
standard deviation of delta error (deg)	7.815	7.283	6.123	6.340

**Table 4:** Aggregated WebGazer performance according to fixed and pseudo-random evaluation-point distribution.

Evaluation Metrics	Fixed	Pseudo-Random
mean spatial error (px)	1009	1160
maximum spatial error (px)	2989	3121
minimum spatial error (px)	58	96
standard deviation of spatial error (px)	617	666
mean delta error (deg)	10.322	11.886
maximum delta error (deg)	30.482	31.768
minimum delta error (deg)	0.591	0.996
standard deviation of delta error (deg)	6.340	6.829

**Table 5:** Aggregated WebGazer performance according to target presentation duration.

Evaluation Metrics	2 s	3 s	4 s	5 s
mean spatial error (px)	1334	1316	1184	1009
maximum spatial error (px)	2941	3113	3045	2989
minimum spatial error (px)	212	151	89	58
standard deviation of spatial error (px)	620	642	640	617
mean delta error (deg)	13.741	13.525	12.164	10.322
maximum delta error (deg)	30.055	31.728	31.037	30.482
minimum delta error (deg)	2.198	1.569	0.923	0.591
standard deviation of delta error (deg)	6.352	6.581	6.577	6.340

**Table 6:** Aggregated WebGazer performance according to stimulus (target) color.

Metrics	Black/Black	Blue/Blue	Brown/Brown	Green/Green	Magenta/Magenta	Red/Red
mean spatial error (px)	956	1013	1033	1041	1026	1007
maximum spatial error (px)	2768	2712	2929	2878	2823	2734
minimum spatial error (px)	60	102	63	86	77	89
standard deviation of spatial error (px)	575	555	615	603	597	570
mean delta error (deg)	9.813	10.403	10.586	10.681	10.528	10.332
maximum delta error(deg)	28.282	27.737	29.947	29.433	28.867	28.010
minimum delta error (deg)	0.621	1.047	0.646	0.884	0.799	0.914
standard deviation of delta error (deg)	5.907	5.700	6.328	6.197	6.139	5.871

**Table 7:** Aggregated WebGazer performance according to stimulus (target) size.

Metrics	10/10	12/12	14/14	16/16	18/18	20/20
mean spatial error (px)	1041	968	977	1010	1022	1009
maximum spatial error (px)	2924	2855	2724	2776	2836	2989
minimum spatial error (px)	61	57	60	80	78	58
standard deviation of spatial error (px)	609	597	560	572	612	617
mean delta error (deg)	10.669	9.922	10.028	10.377	10.497	10.322
maximum delta error (deg)	29.862	29.189	27.887	28.364	28.975	30.482
minimum delta error (deg)	0.628	0.589	0.628	0.818	0.792	0.591
standard deviation of delta error (deg)	6.258	6.149	5.763	5.882	6.289	6.340

## Data Availability

Participant-level summary values supporting the descriptive analyses are provided in Appendix A.

## Appendix A. Participant-Level Descriptive Summaries Underlying Table 3, Table 4, Table 5, Table 6 and Table 7

Table A1, Table A2, Table A3, Table A4 and Table A5 present the participant-level summaries underlying Table 3, Table 4, Table 5, Table 6 and Table 7. Each cell reports spatial error in scale-adjusted device pixels on the first line and angular error δ in degrees on the second, as mean ± SD [minimum–maximum] after the within-run exclusion criterion in Section 3.3. Participants are anonymized as P01–P20. Spatial values are shown as whole device pixels by discarding the fractional component, consistent with Table 3, Table 4, Table 5, Table 6 and Table 7; angular values are shown to three decimals. In Table 3, Table 4, Table 5, Table 6 and Table 7, maximum, minimum, and standard-deviation entries are arithmetic means of participant-level run summaries, not pooled statistics. The shared reference run is repeated in Table A1, Table A2, Table A3 and Table A5 for factor-wise comparison and is not an additional run.

**Table A1:** Participant-level WebGazer performance according to the joint calibration/evaluation point-density configuration.

Participant	5-5	9-9	12-12	18-18
**P01**	1649 ± 856 [278–4185] 15.584 ± 7.866 [2.743–41.654]	1682 ± 922 [84–4512] 16.551 ± 8.943 [0.822–44.714]	1136 ± 610 [30–3128] 11.380 ± 6.121 [0.303–31.924]	974 ± 564 [36–2685] 9.981 ± 5.819 [0.349–27.432]
**P02**	1441 ± 997 [4–4635] 13.508 ± 9.452 [0.031–45.473]	1178 ± 549 [9–2999] 11.845 ± 5.549 [0.095–30.381]	1182 ± 699 [75–3281] 11.847 ± 7.078 [0.780–33.068]	1006 ± 626 [68–2972] 10.311 ± 6.429 [0.712–30.376]
**P03**	1613 ± 833 [293–4270] 15.228 ± 8.067 [2.615–41.032]	1501 ± 838 [112–3872] 15.025 ± 8.416 [1.130–38.902]	924 ± 466 [120–2516] 9.258 ± 4.702 [1.156–25.737]	1059 ± 657 [28–2998] 10.840 ± 6.748 [0.284–30.638]
**P04**	1669 ± 1011 [114–4753] 15.879 ± 9.853 [0.886–46.480]	1622 ± 816 [37–4413] 16.424 ± 8.240 [0.367–43.214]	733 ± 549 [42–2846] 7.426 ± 5.649 [0.437–29.077]	1124 ± 802 [27–3873] 11.480 ± 8.160 [0.264–39.051]
**P05**	1434 ± 888 [40–3840] 14.192 ± 8.823 [0.381–38.066]	1555 ± 958 [196–5350] 15.351 ± 9.144 [1.950–50.693]	1010 ± 672 [87–3142] 10.163 ± 6.876 [0.875–32.089]	899 ± 558 [86–2955] 9.204 ± 5.750 [0.900–30.098]
**P06**	1493 ± 840 [8–4107] 14.191 ± 7.703 [0.074–40.292]	1359 ± 894 [63–3992] 13.536 ± 8.905 [0.624–37.818]	925 ± 575 [104–2667] 9.422 ± 5.913 [1.086–27.292]	820 ± 586 [43–2832] 8.377 ± 6.017 [0.437–29.024]
**P07**	1165 ± 670 [90–3081] 11.631 ± 6.600 [0.946–30.609]	1410 ± 701 [131–3582] 13.950 ± 6.810 [1.298–36.013]	998 ± 673 [23–3246] 10.020 ± 6.847 [0.218–33.098]	996 ± 600 [50–2994] 10.164 ± 6.163 [0.508–30.539]
**P08**	1162 ± 694 [22–3121] 11.288 ± 6.801 [0.231–31.873]	929 ± 666 [4–2958] 9.343 ± 6.741 [0.041–30.242]	996 ± 621 [67–2952] 10.104 ± 6.361 [0.663–30.156]	970 ± 554 [135–2806] 9.946 ± 5.731 [1.320–28.705]
**P09**	1298 ± 601 [162–3290] 12.686 ± 5.882 [1.702–33.541]	1380 ± 827 [82–3971] 13.842 ± 8.248 [0.798–38.323]	949 ± 567 [72–2959] 9.644 ± 5.833 [0.751–30.349]	1055 ± 689 [35–2882] 10.747 ± 7.085 [0.357–29.424]
**P10**	1200 ± 726 [44–3060] 11.677 ± 7.073 [0.422–30.721]	1225 ± 589 [116–3195] 12.329 ± 5.995 [1.219–32.612]	1162 ± 710 [74–3224] 11.837 ± 7.279 [0.706–32.892]	1080 ± 616 [33–2886] 11.053 ± 6.316 [0.345–29.089]
**P11**	1022 ± 664 [4–3557] 10.169 ± 6.731 [0.040–35.949]	1246 ± 745 [152–3591] 12.678 ± 7.621 [1.536–35.838]	957 ± 532 [183–2896] 9.737 ± 5.444 [1.914–29.620]	848 ± 509 [27–2613] 8.694 ± 5.221 [0.273–26.714]
**P12**	1116 ± 815 [49–3843] 10.950 ± 7.895 [0.476–38.326]	1489 ± 912 [112–4360] 14.966 ± 9.114 [1.097–43.382]	1135 ± 602 [45–2920] 11.600 ± 6.179 [0.469–29.850]	1093 ± 777 [45–3449] 11.157 ± 7.937 [0.469–35.066]
**P13**	1275 ± 570 [101–2960] 12.767 ± 5.750 [1.061–30.031]	904 ± 634 [12–3338] 9.064 ± 6.401 [0.119–34.016]	1035 ± 668 [112–3223] 10.562 ± 6.863 [1.151–32.883]	1079 ± 604 [45–3049] 11.046 ± 6.227 [0.471–31.141]
**P14**	1278 ± 732 [29–3479] 12.516 ± 7.253 [0.305–35.344]	878 ± 599 [93–2849] 8.923 ± 6.149 [0.907–29.111]	1091 ± 537 [33–2672] 11.125 ± 5.555 [0.334–27.315]	982 ± 586 [25–2927] 10.058 ± 6.038 [0.250–29.941]
**P15**	1749 ± 957 [37–4095] 17.558 ± 9.561 [0.369–40.072]	1115 ± 540 [108–2763] 11.148 ± 5.475 [1.060–28.409]	1003 ± 522 [102–2685] 10.234 ± 5.341 [1.031–27.443]	1100 ± 631 [123–3133] 11.249 ± 6.514 [1.263–31.999]
**P16**	1451 ± 1065 [51–3866] 14.520 ± 10.548 [0.485–39.000]	885 ± 608 [66–2987] 8.990 ± 6.222 [0.679–30.425]	974 ± 599 [56–2841] 9.905 ± 6.151 [0.586–29.009]	997 ± 647 [57–3276] 10.215 ± 6.657 [0.542–33.378]
**P17**	1525 ± 742 [317–4161] 15.149 ± 7.366 [3.327–41.676]	1044 ± 708 [44–3200] 10.543 ± 7.252 [0.442–32.647]	1087 ± 641 [42–3041] 11.089 ± 6.593 [0.439–31.083]	923 ± 497 [87–2458] 9.454 ± 5.077 [0.911–25.138]
**P18**	1328 ± 767 [69–3493] 13.345 ± 7.669 [0.680–35.193]	1135 ± 827 [37–3534] 11.485 ± 8.465 [0.373–35.852]	1158 ± 580 [25–2661] 11.836 ± 5.986 [0.243–27.213]	1013 ± 609 [114–2977] 10.349 ± 6.226 [1.190–30.411]
**P19**	1424 ± 722 [189–3580] 14.305 ± 7.331 [1.864–34.876]	962 ± 534 [70–2863] 9.693 ± 5.437 [0.736–29.284]	1069 ± 527 [27–2419] 10.930 ± 5.389 [0.282–24.682]	1027 ± 597 [24–3016] 10.451 ± 6.068 [0.250–30.636]
**P20**	1381 ± 794 [54–3468] 13.971 ± 8.079 [0.537–34.819]	964 ± 634 [145–3166] 9.764 ± 6.525 [1.425–32.309]	1229 ± 615 [64–3040] 12.583 ± 6.309 [0.667–31.060]	1140 ± 645 [75–3009] 11.668 ± 6.622 [0.727–30.844]

Note: First line: spatial error (device pixels), second line: angular error δ (degrees). Values are mean ± SD [minimum–maximum], SD = standard deviation. Point-density labels give the matched numbers of calibration and evaluation points, for example 5-5 denotes five calibration and five evaluation points.

**Table A2:** Participant-level WebGazer performance according to evaluation-point distribution.

Participant	Fixed	Pseudo-Random
**P01**	974 ± 564 [36–2685] 9.981 ± 5.819 [0.349–27.432]	933 ± 556 [64–2818] 9.563 ± 5.733 [0.670–28.657]
**P02**	1006 ± 626 [68–2972] 10.311 ± 6.429 [0.712–30.376]	1031 ± 635 [72–2988] 10.591 ± 6.564 [0.730–30.581]
**P03**	1059 ± 657 [28–2998] 10.840 ± 6.748 [0.284–30.638]	1199 ± 670 [82–3011] 12.256 ± 6.838 [0.858–30.587]
**P04**	1124 ± 802 [27–3873] 11.480 ± 8.160 [0.264–39.051]	1261 ± 681 [34–3100] 12.918 ± 6.973 [0.346–31.730]
**P05**	899 ± 558 [86–2955] 9.204 ± 5.750 [0.900–30.098]	984 ± 725 [30–3257] 10.084 ± 7.444 [0.296–33.114]
**P06**	820 ± 586 [43–2832] 8.377 ± 6.017 [0.437–29.024]	1146 ± 647 [151–3111] 11.728 ± 6.633 [1.582–31.197]
**P07**	996 ± 600 [50–2994] 10.164 ± 6.163 [0.508–30.539]	994 ± 573 [103–2738] 10.169 ± 5.903 [1.079–27.976]
**P08**	970 ± 554 [135–2806] 9.946 ± 5.731 [1.320–28.705]	1097 ± 603 [85–2901] 11.268 ± 6.161 [0.888–29.660]
**P09**	1055 ± 689 [35–2882] 10.747 ± 7.085 [0.357–29.424]	1156 ± 660 [167–3023] 11.843 ± 6.772 [1.710–30.948]
**P10**	1080 ± 616 [33–2886] 11.053 ± 6.316 [0.345–29.089]	1164 ± 669 [152–3362] 11.892 ± 6.876 [1.467–34.262]
**P11**	848 ± 509 [27–2613] 8.694 ± 5.221 [0.273–26.714]	1101 ± 609 [21–2800] 11.260 ± 6.261 [0.220–28.666]
**P12**	1093 ± 777 [45–3449] 11.157 ± 7.937 [0.469–35.066]	1096 ± 546 [122–2543] 11.256 ± 5.607 [1.273–26.028]
**P13**	1079 ± 604 [45–3049] 11.046 ± 6.227 [0.471–31.141]	1230 ± 587 [212–3038] 12.653 ± 6.039 [2.189–31.034]
**P14**	982 ± 586 [25–2927] 10.058 ± 6.038 [0.250–29.941]	1217 ± 859 [15–3796] 12.446 ± 8.775 [0.149–38.356]
**P15**	1100 ± 631 [123–3133] 11.249 ± 6.514 [1.263–31.999]	1361 ± 714 [103–3323] 13.948 ± 7.278 [1.056–33.806]
**P16**	997 ± 647 [57–3276] 10.215 ± 6.657 [0.542–33.378]	1434 ± 852 [96–3481] 14.649 ± 8.713 [0.989–35.033]
**P17**	923 ± 497 [87–2458] 9.454 ± 5.077 [0.911–25.138]	1095 ± 720 [16–3436] 11.188 ± 7.312 [0.150–34.943]
**P18**	1013 ± 609 [114–2977] 10.349 ± 6.226 [1.190–30.411]	1447 ± 826 [27–3810] 14.754 ± 8.388 [0.275–38.484]
**P19**	1027 ± 597 [24–3016] 10.451 ± 6.068 [0.250–30.636]	1059 ± 645 [48–2952] 10.879 ± 6.612 [0.494–30.259]
**P20**	1140 ± 645 [75–3009] 11.668 ± 6.622 [0.727–30.844]	1205 ± 552 [333–2934] 12.368 ± 5.693 [3.493–30.048]

Note: First line: spatial error (device pixels), second line: angular error δ (degrees). Values are mean ± SD [minimum–maximum], SD = standard deviation.

**Table A3:** Participant-level WebGazer performance according to target presentation duration.

Participant	2 s	3 s	4 s	5 s
**P01**	1246 ± 668 [140–3232] 12.822 ± 6.837 [1.464–32.978]	1199 ± 577 [61–2864] 12.325 ± 5.905 [0.637–29.286]	1129 ± 650 [24–3088] 11.604 ± 6.695 [0.242–31.543]	974 ± 564 [36–2685] 9.981 ± 5.819 [0.349–27.432]
**P02**	1276 ± 526 [490–2897] 13.142 ± 5.382 [4.893–29.602]	1502 ± 664 [285–3379] 15.359 ± 6.779 [2.893–33.866]	1087 ± 626 [68–2872] 11.154 ± 6.431 [0.711–29.381]	1006 ± 626 [68–2972] 10.311 ± 6.429 [0.712–30.376]
**P03**	1304 ± 606 [229–3163] 13.426 ± 6.214 [2.394–32.294]	1182 ± 589 [37–3103] 12.142 ± 6.073 [0.372–31.679]	1112 ± 556 [77–2680] 11.428 ± 5.717 [0.792–27.419]	1059 ± 657 [28–2998] 10.840 ± 6.748 [0.284–30.638]
**P04**	1268 ± 560 [110–2542] 13.065 ± 5.702 [1.150–25.998]	1219 ± 516 [106–2713] 12.516 ± 5.314 [1.108–27.764]	1333 ± 724 [131–3446] 13.597 ± 7.329 [1.371–34.204]	1124 ± 802 [27–3873] 11.480 ± 8.160 [0.264–39.051]
**P05**	1302 ± 753 [25–3438] 13.394 ± 7.698 [0.261–34.950]	1300 ± 644 [323–2977] 13.360 ± 6.597 [3.293–30.181]	1109 ± 673 [45–3196] 11.385 ± 6.928 [0.446–32.620]	899 ± 558 [86–2955] 9.204 ± 5.750 [0.900–30.098]
**P06**	1393 ± 608 [545–3258] 14.338 ± 6.235 [5.698–33.205]	1261 ± 631 [204–3246] 12.953 ± 6.485 [2.129–33.114]	1083 ± 659 [100–3033] 11.108 ± 6.774 [1.023–30.749]	820 ± 586 [43–2832] 8.377 ± 6.017 [0.437–29.024]
**P07**	1273 ± 687 [90–2838] 13.099 ± 7.026 [0.921–29.037]	1409 ± 544 [278–2703] 14.510 ± 5.587 [2.910–27.796]	1286 ± 758 [55–3468] 13.188 ± 7.783 [0.575–35.271]	996 ± 600 [50–2994] 10.164 ± 6.163 [0.508–30.539]
**P08**	1441 ± 549 [359–2627] 14.855 ± 5.620 [3.749–26.882]	1428 ± 691 [253–3375] 14.674 ± 7.087 [2.647–34.347]	1135 ± 642 [91–3091] 11.631 ± 6.579 [0.951–31.582]	970 ± 554 [135–2806] 9.946 ± 5.731 [1.320–28.705]
**P09**	1315 ± 599 [110–2844] 13.548 ± 6.118 [1.151–29.094]	1587 ± 620 [334–3190] 16.259 ± 6.308 [3.498–32.536]	1090 ± 508 [75–2666] 11.195 ± 5.201 [0.781–27.279]	1055 ± 689 [35–2882] 10.747 ± 7.085 [0.357–29.424]
**P10**	1384 ± 622 [256–2524] 14.203 ± 6.363 [2.678–25.939]	1189 ± 488 [154–2734] 12.218 ± 5.025 [1.608–27.972]	1241 ± 600 [267–3099] 12.767 ± 6.161 [2.752–31.661]	1080 ± 616 [33–2886] 11.053 ± 6.316 [0.345–29.089]
**P11**	1389 ± 656 [226–3331] 14.306 ± 6.693 [2.360–33.947]	1268 ± 641 [281–3185] 13.009 ± 6.550 [2.942–32.350]	1300 ± 589 [152–2917] 13.362 ± 6.030 [1.589–29.826]	848 ± 509 [27–2613] 8.694 ± 5.221 [0.273–26.714]
**P12**	1395 ± 649 [366–3127] 14.366 ± 6.643 [3.622–31.938]	1506 ± 703 [17–3108] 15.479 ± 7.196 [0.175–31.709]	1309 ± 606 [92–2967] 13.466 ± 6.212 [0.914–30.339]	1093 ± 777 [45–3449] 11.157 ± 7.937 [0.469–35.066]
**P13**	1333 ± 588 [156–2771] 13.732 ± 5.990 [1.633–28.324]	1124 ± 628 [102–2943] 11.542 ± 6.431 [1.067–30.080]	1278 ± 555 [72–2712] 13.157 ± 5.719 [0.731–27.752]	1079 ± 604 [45–3049] 11.046 ± 6.227 [0.471–31.141]
**P14**	1303 ± 493 [203–2427] 13.408 ± 5.012 [2.123–24.816]	1432 ± 788 [126–3662] 14.666 ± 8.048 [1.253–37.120]	1145 ± 571 [167–3148] 11.785 ± 5.863 [1.739–32.147]	982 ± 586 [25–2927] 10.058 ± 6.038 [0.250–29.941]
**P15**	1272 ± 545 [91–2925] 13.105 ± 5.563 [0.948–29.915]	1248 ± 685 [135–2989] 12.824 ± 7.006 [1.408–30.530]	1373 ± 622 [123–2958] 14.122 ± 6.353 [1.253–30.205]	1100 ± 631 [123–3133] 11.249 ± 6.514 [1.263–31.999]
**P16**	1323 ± 622 [230–2793] 13.625 ± 6.353 [2.401–28.555]	1278 ± 730 [67–3404] 13.140 ± 7.489 [0.700–34.656]	1111 ± 753 [15–3357] 11.402 ± 7.719 [0.157–34.142]	997 ± 647 [57–3276] 10.215 ± 6.657 [0.542–33.378]
**P17**	1401 ± 739 [138–3008] 14.410 ± 7.580 [1.395–30.752]	1339 ± 691 [40–3229] 13.768 ± 7.076 [0.419–32.938]	1222 ± 718 [83–3210] 12.527 ± 7.394 [0.837–32.747]	923 ± 497 [87–2458] 9.454 ± 5.077 [0.911–25.138]
**P18**	1308 ± 558 [171–2851] 13.482 ± 5.727 [1.787–29.166]	1491 ± 778 [53–3198] 15.324 ± 7.974 [0.553–32.636]	1158 ± 690 [28–2939] 11.870 ± 7.105 [0.293–30.045]	1013 ± 609 [114–2977] 10.349 ± 6.226 [1.190–30.411]
**P19**	1324 ± 713 [129–3002] 13.630 ± 7.310 [1.348–30.691]	1136 ± 576 [111–3049] 11.680 ± 5.901 [1.142–31.160]	1092 ± 614 [66–2703] 11.214 ± 6.342 [0.689–27.661]	1027 ± 597 [24–3016] 10.451 ± 6.068 [0.250–30.636]
**P20**	1449 ± 678 [195–3237] 14.864 ± 6.981 [1.985–33.020]	1240 ± 661 [61–3218] 12.751 ± 6.788 [0.629–32.838]	1103 ± 700 [62–3353] 11.316 ± 7.199 [0.613–34.158]	1140 ± 645 [75–3009] 11.668 ± 6.622 [0.727–30.844]

Note: First line: spatial error (device pixels), second line: angular error δ (degrees). Values are mean ± SD [minimum–maximum], SD = standard deviation.

**Table A4:** Participant-level WebGazer performance according to matched calibration/evaluation target color.

Participant	Black/Black	Blue/Blue	Brown/Brown	Green/Green	Magenta/Magenta	Red/Red
**P01**	844 ± 605 [45–2841] 8.658 ± 6.230 [0.462–29.067]	959 ± 588 [3–2947] 9.862 ± 6.074 [0.031–30.129]	1090 ± 663 [3–3040] 11.190 ± 6.820 [0.031–31.051]	1013 ± 584 [152–2422] 10.399 ± 5.996 [1.518–25.027]	1010 ± 634 [23–2910] 10.359 ± 6.516 [0.241–29.753]	947 ± 576 [42–2703] 9.707 ± 5.936 [0.430–27.650]
**P02**	894 ± 533 [68–2733] 9.176 ± 5.486 [0.710–27.965]	1007 ± 732 [28–3178] 10.328 ± 7.543 [0.285–32.444]	1027 ± 628 [23–2961] 10.507 ± 6.445 [0.229–30.274]	1098 ± 669 [111–2946] 11.251 ± 6.845 [1.108–30.101]	1060 ± 590 [6–2626] 10.855 ± 6.067 [0.063–26.853]	1011 ± 583 [48–2709] 10.357 ± 5.997 [0.502–27.813]
**P03**	942 ± 623 [44–3040] 9.651 ± 6.396 [0.435–30.880]	970 ± 551 [112–2671] 9.955 ± 5.656 [1.110–27.334]	1010 ± 648 [85–3189] 10.340 ± 6.655 [0.862–32.552]	1090 ± 601 [72–2770] 11.179 ± 6.167 [0.740–28.334]	1277 ± 788 [58–3486] 13.047 ± 8.046 [0.554–35.448]	1108 ± 594 [201–3021] 11.360 ± 6.097 [2.083–30.804]
**P04**	940 ± 596 [85–2837] 9.646 ± 6.160 [0.863–29.027]	987 ± 516 [94–2820] 10.136 ± 5.304 [0.943–28.842]	1142 ± 550 [69–2766] 11.725 ± 5.662 [0.721–28.331]	1103 ± 634 [55–2904] 11.310 ± 6.494 [0.573–29.630]	1059 ± 634 [36–3125] 10.822 ± 6.511 [0.352–31.898]	918 ± 537 [68–2585] 9.402 ± 5.519 [0.701–26.632]
**P05**	899 ± 557 [25–2703] 9.224 ± 5.722 [0.257–27.657]	1025 ± 538 [197–2650] 10.525 ± 5.515 [2.061–27.119]	1003 ± 669 [23–2686] 10.259 ± 6.857 [0.240–27.379]	1060 ± 569 [64–2867] 10.871 ± 5.871 [0.668–29.419]	1076 ± 607 [132–2788] 11.033 ± 6.243 [1.380–28.660]	954 ± 584 [85–2789] 9.766 ± 6.003 [0.876–28.536]
**P06**	928 ± 590 [26–2816] 9.515 ± 6.059 [0.262–28.806]	1048 ± 602 [36–2622] 10.758 ± 6.195 [0.362–26.796]	949 ± 636 [45–3003] 9.704 ± 6.517 [0.471–30.658]	1106 ± 486 [159–2614] 11.361 ± 4.998 [1.664–26.718]	1071 ± 647 [102–2693] 11.002 ± 6.652 [1.068–27.537]	1060 ± 669 [45–3031] 10.861 ± 6.875 [0.455–30.975]
**P07**	854 ± 591 [66–2681] 8.757 ± 6.082 [0.668–27.423]	963 ± 591 [141–2577] 9.888 ± 6.080 [1.475–26.557]	1156 ± 681 [66–3211] 11.813 ± 7.013 [0.666–32.749]	1160 ± 691 [51–3226] 11.885 ± 7.068 [0.516–32.845]	927 ± 618 [75–2885] 9.517 ± 6.373 [0.758–29.513]	987 ± 546 [197–2606] 10.121 ± 5.613 [1.992–26.806]
**P08**	1048 ± 760 [33–3279] 10.735 ± 7.786 [0.345–33.439]	1172 ± 516 [219–2869] 12.046 ± 5.297 [2.164–29.351]	1024 ± 572 [85–2762] 10.510 ± 5.880 [0.823–28.258]	1104 ± 623 [217–3328] 11.333 ± 6.388 [2.205–33.878]	999 ± 491 [72–2574] 10.253 ± 5.035 [0.739–26.327]	943 ± 534 [82–2628] 9.681 ± 5.502 [0.857–26.956]
**P09**	903 ± 518 [6–2538] 9.262 ± 5.329 [0.058–25.950]	977 ± 546 [100–2711] 10.049 ± 5.597 [1.046–27.741]	985 ± 506 [115–2607] 10.100 ± 5.213 [1.162–26.662]	1125 ± 624 [65–3060] 11.565 ± 6.423 [0.679–31.268]	1009 ± 606 [42–2872] 10.339 ± 6.234 [0.432–29.383]	922 ± 568 [36–2499] 9.447 ± 5.847 [0.376–25.549]
**P10**	869 ± 586 [37–2578] 8.921 ± 6.039 [0.381–26.377]	1119 ± 518 [307–2703] 11.495 ± 5.323 [3.215–27.650]	1093 ± 628 [49–3038] 11.214 ± 6.449 [0.469–30.997]	955 ± 657 [39–2865] 9.787 ± 6.740 [0.407–29.303]	1059 ± 608 [62–2947] 10.851 ± 6.262 [0.648–30.129]	967 ± 560 [77–2767] 9.907 ± 5.772 [0.804–28.426]
**P11**	940 ± 506 [44–2574] 9.638 ± 5.192 [0.460–26.337]	1135 ± 423 [164–2543] 11.669 ± 4.307 [1.715–25.991]	967 ± 651 [158–3114] 9.881 ± 6.689 [1.576–31.785]	943 ± 578 [15–2789] 9.660 ± 5.950 [0.157–28.534]	928 ± 550 [109–2467] 9.510 ± 5.653 [1.141–25.230]	1002 ± 604 [82–2925] 10.261 ± 6.216 [0.858–29.902]
**P12**	1023 ± 555 [94–2657] 10.491 ± 5.688 [0.977–27.183]	914 ± 498 [52–2539] 9.374 ± 5.100 [0.543–25.966]	1069 ± 599 [43–3030] 10.911 ± 6.173 [0.439–30.986]	1046 ± 630 [128–3086] 10.716 ± 6.477 [1.279–31.527]	981 ± 536 [60–2808] 10.074 ± 5.516 [0.617–28.732]	947 ± 591 [125–2742] 9.709 ± 6.099 [1.212–28.058]
**P13**	1089 ± 626 [48–3019] 11.146 ± 6.411 [0.502–30.483]	1028 ± 578 [19–2740] 10.548 ± 5.945 [0.199–28.018]	928 ± 555 [64–2779] 9.506 ± 5.695 [0.668–28.474]	926 ± 553 [114–2594] 9.495 ± 5.694 [1.137–26.540]	1002 ± 563 [119–2756] 10.282 ± 5.804 [1.245–28.198]	965 ± 562 [36–2832] 9.895 ± 5.766 [0.375–28.974]
**P14**	990 ± 511 [42–2637] 10.162 ± 5.241 [0.437–26.985]	1024 ± 518 [136–2312] 10.510 ± 5.267 [1.379–23.615]	917 ± 580 [51–2791] 9.405 ± 5.978 [0.533–28.555]	1013 ± 632 [26–2988] 10.362 ± 6.482 [0.272–30.544]	1044 ± 584 [91–2795] 10.724 ± 5.991 [0.947–28.570]	928 ± 580 [60–2562] 9.512 ± 5.978 [0.577–26.272]
**P15**	968 ± 413 [252–2286] 9.944 ± 4.227 [2.636–23.330]	974 ± 639 [96–2855] 9.987 ± 6.560 [1.003–29.089]	1029 ± 572 [86–2952] 10.489 ± 5.891 [0.869–30.288]	951 ± 589 [45–2699] 9.762 ± 6.066 [0.471–27.620]	1034 ± 524 [211–2614] 10.621 ± 5.405 [2.205–26.747]	1012 ± 592 [114–2740] 10.395 ± 6.103 [1.133–28.035]
**P16**	1057 ± 548 [126–2701] 10.828 ± 5.628 [1.237–27.770]	996 ± 616 [102–2946] 10.152 ± 6.298 [1.013–30.231]	942 ± 567 [46–2919] 9.677 ± 5.838 [0.480–29.822]	1039 ± 646 [21–3155] 10.671 ± 6.654 [0.220–32.218]	994 ± 613 [27–2999] 10.210 ± 6.318 [0.278–30.593]	1068 ± 557 [39–2778] 10.966 ± 5.728 [0.400–28.409]
**P17**	1068 ± 710 [33–3258] 10.970 ± 7.311 [0.345–33.227]	946 ± 528 [30–2624] 9.695 ± 5.427 [0.312–26.836]	955 ± 645 [43–2879] 9.801 ± 6.654 [0.450–29.426]	945 ± 593 [32–2865] 9.685 ± 6.113 [0.306–29.297]	979 ± 572 [47–2704] 10.039 ± 5.873 [0.491–27.729]	1166 ± 653 [119–3008] 11.974 ± 6.718 [1.245–30.747]
**P18**	961 ± 510 [82–2645] 9.858 ± 5.229 [0.831–27.061]	965 ± 564 [85–2708] 9.896 ± 5.811 [0.889–27.712]	1106 ± 624 [13–2691] 11.348 ± 6.394 [0.133–27.676]	1032 ± 575 [88–2736] 10.591 ± 5.895 [0.900–27.992]	951 ± 562 [95–2903] 9.768 ± 5.777 [0.989–29.675]	1030 ± 452 [174–2366] 10.575 ± 4.613 [1.790–24.385]
**P19**	925 ± 614 [45–3018] 9.475 ± 6.301 [0.458–30.795]	1028 ± 538 [80–2621] 10.559 ± 5.509 [0.787–26.658]	1080 ± 639 [57–2899] 11.072 ± 6.559 [0.595–29.582]	993 ± 583 [185–2827] 10.144 ± 5.965 [1.906–28.956]	1038 ± 637 [30–2905] 10.648 ± 6.545 [0.312–29.696]	1148 ± 581 [88–2891] 11.791 ± 5.965 [0.885–29.570]
**P20**	995 ± 550 [9–2530] 10.209 ± 5.620 [0.094–25.879]	1036 ± 506 [41–2605] 10.634 ± 5.186 [0.411–26.654]	1199 ± 699 [146–3280] 12.259 ± 7.179 [1.494–33.439]	1132 ± 551 [96–2819] 11.595 ± 5.647 [0.953–28.916]	1033 ± 581 [150–2606] 10.601 ± 5.962 [1.525–26.666]	1065 ± 492 [69–2515] 10.943 ± 5.073 [0.722–25.699]

Note: First line: spatial error (device pixels), second line: angular error δ (degrees). Values are mean ± SD [minimum–maximum], SD = standard deviation. Color pairs identify the calibration and evaluation target colors, respectively.

**Table A5:** Participant-level WebGazer performance according to calibration/evaluation target diameter.

Participant	10/10	12/12	14/14	16/16	18/18	20/20
**P01**	1127 ± 638 [51–3147] 11.527 ± 6.539 [0.522–31.962]	991 ± 537 [88–2485] 10.177 ± 5.537 [0.905–25.383]	1004 ± 506 [55–2545] 10.310 ± 5.185 [0.575–26.038]	1097 ± 532 [64–2650] 11.277 ± 5.467 [0.658–27.111]	995 ± 639 [39–2813] 10.209 ± 6.556 [0.407–28.782]	974 ± 564 [36–2685] 9.981 ± 5.819 [0.349–27.432]
**P02**	1094 ± 615 [82–3091] 11.201 ± 6.324 [0.842–31.591]	874 ± 570 [34–2781] 8.955 ± 5.876 [0.350–28.455]	961 ± 487 [66–2591] 9.868 ± 5.011 [0.690–26.510]	1141 ± 619 [47–2779] 11.726 ± 6.361 [0.468–28.477]	1059 ± 617 [170–2930] 10.869 ± 6.342 [1.723–29.884]	1006 ± 626 [68–2972] 10.311 ± 6.429 [0.712–30.376]
**P03**	920 ± 611 [30–2883] 9.420 ± 6.307 [0.303–29.607]	1036 ± 651 [112–3159] 10.619 ± 6.684 [1.149–32.255]	1060 ± 617 [52–2876] 10.888 ± 6.354 [0.535–29.414]	1052 ± 568 [112–2924] 10.775 ± 5.828 [1.086–29.900]	944 ± 610 [123–2951] 9.665 ± 6.273 [1.251–30.238]	1059 ± 657 [28–2998] 10.840 ± 6.748 [0.284–30.638]
**P04**	1097 ± 638 [35–3094] 11.239 ± 6.547 [0.365–31.592]	890 ± 568 [56–2797] 9.133 ± 5.856 [0.552–28.612]	1027 ± 565 [130–2829] 10.539 ± 5.776 [1.360–28.936]	873 ± 514 [42–2535] 8.965 ± 5.307 [0.438–25.934]	962 ± 645 [42–2935] 9.863 ± 6.627 [0.439–29.966]	1124 ± 802 [27–3873] 11.480 ± 8.160 [0.264–39.051]
**P05**	1059 ± 585 [33–2962] 10.840 ± 5.985 [0.321–30.249]	922 ± 616 [54–3045] 9.446 ± 6.353 [0.553–31.122]	939 ± 526 [46–2730] 9.637 ± 5.415 [0.481–27.930]	903 ± 619 [39–2762] 9.263 ± 6.362 [0.378–28.241]	974 ± 612 [6–2788] 10.005 ± 6.301 [0.063–28.529]	899 ± 558 [86–2955] 9.204 ± 5.750 [0.900–30.098]
**P06**	1195 ± 725 [71–3234] 12.219 ± 7.432 [0.742–32.733]	1002 ± 603 [64–2737] 10.252 ± 6.196 [0.666–27.990]	999 ± 510 [6–2545] 10.250 ± 5.239 [0.063–26.240]	987 ± 556 [120–2626] 10.138 ± 5.720 [1.223–26.870]	921 ± 629 [40–2954] 9.458 ± 6.490 [0.411–30.207]	820 ± 586 [43–2832] 8.377 ± 6.017 [0.437–29.024]
**P07**	1005 ± 653 [75–3058] 10.293 ± 6.696 [0.761–31.178]	877 ± 515 [63–2752] 8.993 ± 5.295 [0.659–28.086]	942 ± 582 [71–2801] 9.666 ± 5.990 [0.743–28.641]	933 ± 557 [36–2622] 9.573 ± 5.718 [0.377–26.710]	904 ± 555 [33–2583] 9.267 ± 5.725 [0.344–26.625]	996 ± 600 [50–2994] 10.164 ± 6.163 [0.508–30.539]
**P08**	996 ± 601 [89–2935] 10.197 ± 6.190 [0.916–30.018]	933 ± 565 [15–2433] 9.586 ± 5.833 [0.157–24.903]	1020 ± 568 [90–2795] 10.468 ± 5.846 [0.908–28.582]	1192 ± 684 [52–3253] 12.258 ± 7.049 [0.529–33.166]	924 ± 569 [95–2809] 9.483 ± 5.865 [0.993–28.742]	970 ± 554 [135–2806] 9.946 ± 5.731 [1.320–28.705]
**P09**	927 ± 595 [85–2747] 9.517 ± 6.135 [0.887–28.075]	989 ± 582 [33–2890] 10.148 ± 5.990 [0.324–29.562]	1049 ± 592 [93–2895] 10.747 ± 6.100 [0.971–29.612]	973 ± 581 [140–2585] 9.985 ± 5.990 [1.463–26.450]	1052 ± 670 [59–2828] 10.813 ± 6.873 [0.616–28.932]	1055 ± 689 [35–2882] 10.747 ± 7.085 [0.357–29.424]
**P10**	991 ± 532 [72–2704] 10.187 ± 5.482 [0.753–27.671]	999 ± 502 [178–2468] 10.251 ± 5.180 [1.860–25.241]	986 ± 543 [34–2631] 10.124 ± 5.594 [0.356–26.919]	1144 ± 559 [115–2878] 11.750 ± 5.731 [1.183–29.435]	1068 ± 530 [141–2788] 10.971 ± 5.440 [1.427–28.527]	1080 ± 616 [33–2886] 11.053 ± 6.316 [0.345–29.089]
**P11**	1157 ± 557 [75–2647] 11.882 ± 5.699 [0.769–27.055]	1045 ± 691 [35–3340] 10.661 ± 7.085 [0.353–34.021]	1017 ± 546 [105–2430] 10.440 ± 5.608 [1.095–25.099]	1041 ± 556 [145–3078] 10.696 ± 5.739 [1.423–31.450]	1122 ± 588 [84–2805] 11.521 ± 6.027 [0.860–28.696]	848 ± 509 [27–2613] 8.694 ± 5.221 [0.273–26.714]
**P12**	1132 ± 557 [36–2648] 11.620 ± 5.739 [0.376–27.096]	901 ± 581 [15–2821] 9.225 ± 5.976 [0.154–28.864]	862 ± 548 [32–2509] 8.845 ± 5.652 [0.334–25.658]	957 ± 558 [82–2846] 9.791 ± 5.725 [0.853–28.697]	1179 ± 622 [66–2948] 12.099 ± 6.396 [0.665–30.122]	1093 ± 777 [45–3449] 11.157 ± 7.937 [0.469–35.066]
**P13**	1101 ± 599 [67–2810] 11.307 ± 6.149 [0.698–28.710]	991 ± 590 [21–2742] 10.147 ± 6.065 [0.219–28.060]	932 ± 617 [64–2951] 9.546 ± 6.354 [0.666–30.170]	1070 ± 520 [104–2711] 10.988 ± 5.337 [1.082–27.731]	1093 ± 613 [186–2922] 11.226 ± 6.251 [1.908–29.870]	1079 ± 604 [45–3049] 11.046 ± 6.227 [0.471–31.141]
**P14**	1196 ± 624 [115–2955] 12.225 ± 6.432 [1.197–30.196]	920 ± 609 [12–3088] 9.438 ± 6.267 [0.118–31.541]	948 ± 533 [93–2681] 9.712 ± 5.474 [0.939–27.435]	1126 ± 625 [129–2976] 11.568 ± 6.405 [1.340–30.341]	1068 ± 668 [123–2786] 10.972 ± 6.882 [1.205–28.518]	982 ± 586 [25–2927] 10.058 ± 6.038 [0.250–29.941]
**P15**	942 ± 605 [55–2863] 9.640 ± 6.222 [0.557–29.170]	1111 ± 690 [63–2965] 11.329 ± 7.105 [0.658–30.304]	912 ± 513 [30–2476] 9.346 ± 5.270 [0.291–25.325]	1032 ± 518 [24–2592] 10.604 ± 5.310 [0.250–26.514]	987 ± 548 [121–2558] 10.134 ± 5.631 [1.161–26.164]	1100 ± 631 [123–3133] 11.249 ± 6.514 [1.263–31.999]
**P16**	973 ± 721 [10–3400] 9.892 ± 7.335 [0.101–34.573]	993 ± 562 [112–2969] 10.180 ± 5.791 [1.168–30.329]	951 ± 568 [3–2684] 9.759 ± 5.858 [0.031–27.464]	1068 ± 605 [94–2688] 10.959 ± 6.221 [0.955–27.495]	1030 ± 566 [118–2724] 10.581 ± 5.808 [1.198–27.832]	997 ± 647 [57–3276] 10.215 ± 6.657 [0.542–33.378]
**P17**	938 ± 600 [45–2707] 9.601 ± 6.166 [0.469–27.821]	840 ± 670 [33–2994] 8.591 ± 6.892 [0.344–30.611]	1037 ± 674 [18–3097] 10.611 ± 6.903 [0.175–31.640]	895 ± 519 [104–2510] 9.176 ± 5.328 [1.045–25.676]	967 ± 561 [30–2575] 9.921 ± 5.759 [0.305–26.344]	923 ± 497 [87–2458] 9.454 ± 5.077 [0.911–25.138]
**P18**	1027 ± 591 [118–2834] 10.526 ± 6.094 [1.229–28.927]	1074 ± 572 [43–2749] 11.025 ± 5.880 [0.448–28.132]	911 ± 559 [36–2676] 9.340 ± 5.756 [0.375–27.380]	925 ± 522 [67–2536] 9.500 ± 5.374 [0.652–25.926]	1139 ± 679 [34–3025] 11.650 ± 6.932 [0.346–30.019]	1013 ± 609 [114–2977] 10.349 ± 6.226 [1.190–30.411]
**P19**	1060 ± 593 [12–3013] 10.827 ± 6.078 [0.123–30.777]	1078 ± 670 [66–3210] 11.010 ± 6.879 [0.646–32.760]	898 ± 529 [87–2760] 9.215 ± 5.454 [0.908–28.242]	955 ± 631 [46–2956] 9.805 ± 6.497 [0.479–30.219]	1092 ± 670 [40–3078] 11.212 ± 6.879 [0.389–31.453]	1027 ± 597 [24–3016] 10.451 ± 6.068 [0.250–30.636]
**P20**	901 ± 543 [64–2763] 9.225 ± 5.610 [0.636–28.235]	909 ± 606 [51–2694] 9.277 ± 6.235 [0.502–27.556]	1101 ± 628 [101–2986] 11.243 ± 6.421 [1.057–30.506]	853 ± 600 [45–3029] 8.748 ± 6.165 [0.470–30.931]	978 ± 654 [12–2939] 10.023 ± 6.719 [0.125–30.055]	1140 ± 645 [75–3009] 11.668 ± 6.622 [0.727–30.844]

Note: First line: spatial error (device pixels), second line: angular error δ (degrees). Values are mean ± SD [minimum–maximum], SD = standard deviation. Size pairs identify the calibration and evaluation target size in CSS pixels. Spatial-error values are reported in device pixels.
